# Data set for solar flare prediction using helioseismic and magnetic imager vector magnetic field data

**DOI:** 10.1016/j.dib.2021.107203

**Published:** 2021-06-09

**Authors:** Alciomar Hollanda, Ana Estela Antunes Da Silva, Tiago Cinto

**Affiliations:** a School of Technology - FT, University of Campinas - UNICAMP, Limeira, SP, Brazil; b Adventist University Center of São Paulo – UNASP-HT, Hortolândia, SP, Brazil

**Keywords:** Space weather, Solar flares, Data set of magnetic attributes, Solar events

## Abstract

It is known that solar flares can affect the near-Earth space, incurring in consequences for radio communications. Therefore, there is a need to research systems for monitoring solar events. This article presents a data set which can be used in the analysis of such events. This data set originated from a set of records from magnetic attributes and solar flare data. In order to create this data set, authors used the SunPy library which provided access to data from the Joint Science Operations Center (JSOC) and Space Weather Prediction Center (SWPC). By integrating data from those two sources, 8,874 samples were obtained comprehending the period between May, 2010 and December, 2019. The collected data were stored as a CSV data set. This data set can be used to support the research of solar flare forecasting, as well as to be compared to other data sets or expanded with new attributes.

## Specifications Table

**Table:** 

Subject	Astronomy and Astrophysics
Specific subject area	Solar Flare Data
Type of data	Text files in Comma-Separated Value (CSV) format.
How data were acquired	We collected data through the Python's SunPy library.
Data format	Raw and Processed
Parameters for data collection	Our data comprehend solar flares occurring only within ±70° from the Sun's central meridian. The period in which we sampled the satellites’ data set refers to May 01, 2010 until December 31, 2019.
Description of data collection	We gathered data of solar events from the SunPy library using the module *sunpy.instr.goes*. This module provides a list of GOES events with data comprehending the start and end time of an event, as well as the occurred flare class. Then, once more, we used the SunPy library, but employing its drms module. Such module provides an interface for Python language to access Helioseismic and Magnetic Imager (HMI) data stored by the JSOC, namely the *hmi.sharp* and *cgem.Lorentz* data repositories.
Data source location	Institution: Joint Science Operations Center (JSOC) and Space Weather Prediction Center (SWPC) Country: United States of America Data source: Parameterizations of the solar photospheric magnetic field Primary data sources: JSOC (http://jsoc.stanford.edu/) and SWPC (ftp://ftp.swpc.noaa.gov/pub/warehouse/)
Data accessibility	Repository name: Zenodo Direct URL to dataset: https://doi.org/10.5281/zenodo.4603412 Direct URL to code: https://doi.org/10.5281/zenodo.4603369

## Value of the Data

• Solar flares can affect the near-Earth space, incurring in possible damages for radio communications, satellites, cables for transmitting energy and GPS systems. Thus, there is a need to improve systems’ performance for monitoring those events. This article provides a magnetic field data set, essentially designed to be used by solar flare forecasting systems, which can predict solar flare occurrences.
• Researchers of Artificial Intelligence and Astrophysics can use our magnetic field data to analyze the occurrence of solar flares.
• Current data can be used by flare forecasting systems without any modification, as well as can be updated by including new attributes.

## 1 Data Description

In this article, we provided data in the CSV format. Each record of the final data set corresponds to a solar flare event containing magnetic measures of the last 24 hours. The features of each record are explained in Table 1. The final data set contains 8,874 records: 8,493 non-flare (95,70%) and 381 flare samples (4,30%).

**Table 1:** Features of each record of the final dataset.

Attribute's Name	Description
FLARE_NUMBER	Refers to data from the GOES Event representing whether a flare occurred or not. Attribute's values labeled as 1 are related to M- or X-class flare events. On the other hand, when their values equal 0, they are related to A-, B-, or C-class events, or no event.
T_REC	Contains date and time that the magnetic data were collected from SHARP.
NOAA_AR	Shows the number of the active region where the event occurred (where the magnetic data were taken from).
QUALITY	This attribute refers to a flag from the SHARP's data set showing whether records are noisy (This attribute holds values from a pre-defined table as disposed in [10]). When errors occur during the SHARP's data processing, the quality attribute reports them by holding values higher than 65,536 (or 10,000 in hexadecimal) [1,^3^,^4^,8]. If attribute's values range between 0 and 65,536, their associated data are of good quality. Each value corresponds to a distinct type of error that may occur while processing satellite's data.
LONGITUDE	This attribute was obtained from the SRS data set aiming to perform a filter on the active regions that were outside a defined radius from the central meridian [1,^4^,9]. This attribute shows the longitude in which the active region can be encountered in the solar surface.
LATITUDE	This attribute contains the latitude at which the active region can be found on the solar surface.
TOTUSJH	Total unsigned current helicity. This attribute and all the twenty four following attributes are the data from the Spaceweather HMI Active Region Patch (SHARP) data sets provided by the JSOC. They correspond to magnetic measurements and physical parameters derived from active regions that were automatically tracked by the HMI equipment. Details about those attributes can be found in Bobra [4].
TOTBSQ	Total magnitude of Lorentz force.
TOTPOT	Total photospheric magnetic free energy density.
TOTUSJZ	Total unsigned vertical current.
ABSNJZH	Absolute value of the net current helicity.
SAVNCPP	Sum of the absolute value of the net current per polarity.
USFLUX	Total unsigned flux.
AREA_ACR	Area of strong field pixels in the active region.
TOTFZ	Sum of z-component of Lorentz force.
MEANPOT	Mean photospheric magnetic free energy.
R_VALUE	Sum of flux near polarity inversion line.
EPSZ	Sum of z-component of normalized Lorentz force.
SHRGT45	Fraction of Area with shear >45°.
MEANSHR	Mean shear angle
MEANGAM	Mean angle of field from radial
MEANGBT	Mean gradient of total field
MEANGBZ	Mean gradient of vertical field
MEANGBH	Mean gradient of horizontal field
MEANJZH	Mean current helicity (Bz contribution)
TOTFY	Sum of y-component of Lorentz force
MEANJZD	Mean vertical current density
MEANALP	Mean characteristic twist parameter, α
TOTFX	Sum of x-component of Lorentz force
EPSY	Sum of y-component of normalized Lorentz force
EPSX	Sum of x-component of normalized Lorentz force

Noteworthily, SHARP data are recorded on a daily basis every 12 minutes for each AR. For data reduction purposes, we did not use the mean or median. Instead, to represent positive events (ARs flaring >= M-class flares), we sought the corresponding SHARP data 24 h before the flare occurrence. To identify when an active region triggers a positive event, we employed NOAA's Events data. On the other hand, for negative events, we collected all non-flaring ARs’ (absence of events or < M-class flares) corresponding data at 11:48 PM. Similarly, [4] and [1] research reported to have used similar approaches for assembling their data.

From the data set created, we provide a 5-fold-based test splitting. In this sense, we provided the following groups of training/test sets based on our samples’ years:

• 2010-2011 for test; 2012-2019 for training.
• 2012-2013 for test; 2010-2011 and 2014-2019 for training.
• 2014-2015 for test; 2010-2013 and 2016-2019 for training.
• 2016-2017 for test; 2010-2015 and 2018-2019 for training.
• 2018-2019 for test; 2010-2017 for training.

## 2 Experimental Design, Materials and Methods

This section presents the procedures used to collect data, as well as the definition of positive and negative classes regarding the problem of forecasting solar flares. Besides, we discuss how we integrated and preprocessed (i.e., missing samples removal and data standardization) our data from distinct sources.

### 2.1 Data sources and attribute selection

We used four data sources to assemble data presented in this article, namely: the Sunspot Region Summary (SRS) and GOES Event, both from the Space Weather Prediction Center (SWPC) [6], and both *hmi.sharp_720s* and *cgem.lorentz*, from the JSOC [3]. In particular, in order to form the SHARP data set, we perform an integration between *hmi.sharp_720s* and *cgem.lorentz*. The union between these data sets occurred through the date and time attributes (T _REC, in Table 1) and number of the active region (NOAA _AR, in Table 1).

Data were collected using the Python's [12] SunPy library and processed by the version 2.0.1 of the SunPy open source software package [7]. For data from the GOES Event, we used the Sunpy.instr.goes module. We used the Drms module for data from the SHARP and Sunpy.io.special module for data from the SRS.

**Fig. 1:**
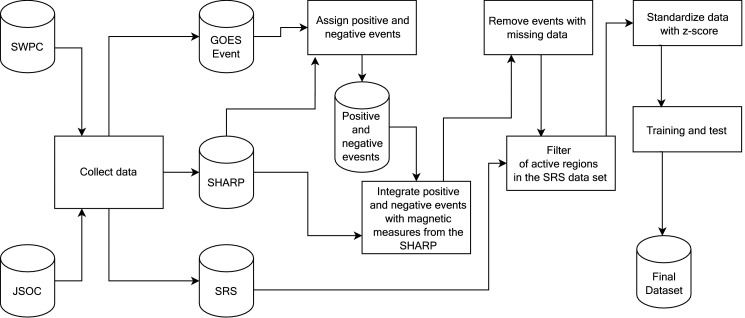
Methodology to assemble data and create the final data set.

All attributes are available in Table 1 and the source of each attribute is shown in Fig. 1.

### 2.2 Data collection procedure

To create our data set, we carried out a five step-based methodology as presented in Fig. 1.

**Fig. 2:**
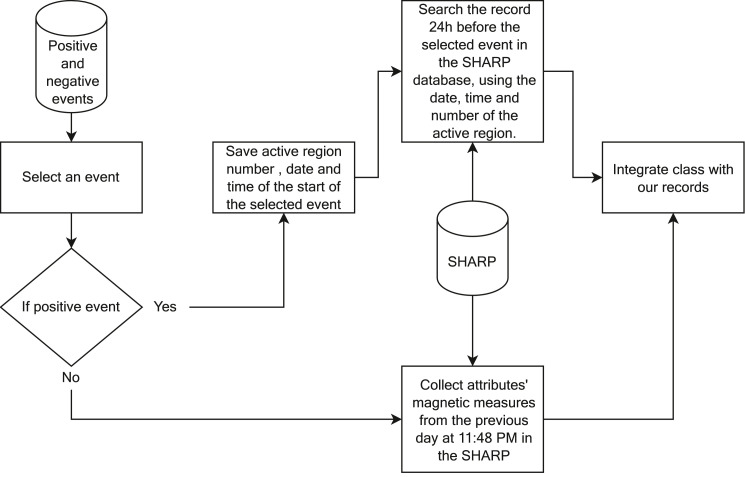
Methodology to integrate data sets.

1. **Collect data:** This module collects data from the SWPC's data sets (GOES Event and SRS data sets) and SHARP data sets from the JSOC's data sets using the Python's SunPy library. The period we collected the data comprised the years between May 2010 and December 2019.
2. **Assign positive and negative events:** This module verifies in GOES Event data, if an active region, flares one M- or X-class event within 24 hours. If the answer is affirmative, then the module assigns the event as belonging to the positive class (label 1). On the other hand, when analyzing SHARP data in 24 hours, active regions that have not had an event reported as M- or X-Class on the GOES Event data, the module assigns the active region as belonging to a negative class event (label 0). It is worth mentioning that an active region flaring more than one event in one day, led us to count them as several distinct positive events. We follow the definition outlined in Bobra [4] and Ahmed [5]. After assigning positive and negative events, the data is stored in a “Positive and negative events” data set so that integration with the magnetic data in the Sharp data set can be done.
3. **Integrate positive and negative events with magnetic measures from the SHARP:** This module presents the integration between the data sets of positive and negative events with the magnetic data attributes, describing the steps to perform the data integration. Fig. 2 contains a representation of these steps. Follow, each step is explained.
  I. Select an event: After the module “Assign positive and negative events”, each event is selected from the data set "Positive and negative events" to be integrated with the magnetic data (SHARP data set).
  II. If the event is positive:
    a. The step: “Save active region number, date and time of the start of the selected event” is chosen. From the attributes contained in the GOES Event data set, only the number of the active region, date and start time of the event are selected. With these attributes, it is possible to search the SHARP data set and identify the magnetic attributes related to the active region that caused a positive event. Some flares in the GOES Event data set are not associated with an active region. We did not include those flares in our data.
    b. The next step is then: “Search the record 24h before the selected event in the SHARP database, using the date, time and number of the active region”. For each positive event (M- or X-class) in the GOES Event data set, we collect the magnetic measures from the SHARP recorded 24 hours before such event.
  III. If the event is negative, the step “Collect attributes' magnetic measures from the previous day at 11:48 PM in the SHARP” is performed. For negative events, only the number of the active region was used to search the SHARP data set. For each active region, the last record of magnetic attributes from the previous day was collected. The last record available in SHARP per day is at 11:48 PM.
  IV. Finally, the step “Integrate class with our records” is performed. After collecting the magnetic attributes for the active region, the type of event is integrated with the magnetic attributes.

It is important to mention that magnetic data are normally available every 12 minutes for each active region. However, there are some cases that the data are not available exactly 24h before a positive and negative event, i.e., 12 minutes before the 24-hour search period. For this reason, there may be magnetic data with an interval greater than 24h in the integration data set.

1. **Removal of events with missing data:** We removed samples from our data set if they had any of their attributes missing measures considering a 24-hour period prior to their associated events. In addition, we also took samples from active regions that reported having noise measurements. The noise is presented in the quality attribute of the magnetic data when errors occur in the processing of SHARP data. We removed all samples that had a quality value higher than 65,536 (or 10,000 in hexadecimal) [4,8].
2. **Filter of active regions in the SRS data set:** This module filters the location of the active region associated with the event (Positive or Negative) in the SRS data set. According to Liu and Bobra [1,4,9], the active regions that are from ±70° show an increase in noise in their magnetic data. For this reason, we filter the active regions that were located on the central meridian of the Sun ±70°. To perform this filter, it was necessary to use the attributes: longitude of the active region (In SRS data set), number of the active region and date (In SRS, SHARP, GOES Event data set).
3. **Standardize data with z-score:** This module standardizes the resulting data using a z-score-based method Han [2] and Nishizuka [11].
4. **Training and test**: In this module we executed a 5-fold-based test splitting. In this sense, we provided the following groups of train/test sets based on our samples’ years:
  • 2010-2011 for test; 2012-2019 for training.
  • 2012-2013 for test; 2010-2011 and 2014-2019 for training.
  • 2014-2015 for test; 2010-2013 and 2016-2019 for training.
  • 2016-2017 for test; 2010-2015 and 2018-2019 for training.
  • 2018-2019 for test; 2010-2017 for training.

## Declaration of Competing Interest

The authors declare that they have no known competing financial interests or personal relationships which have, or could be perceived to have, influenced the work reported in this article.

## Data Availability

DATA-SET-FOR-SOLAR-FLARE-PREDICTION (Original data) (GitHub).
